# Metformin and high-sensitivity cardiac troponin I and T trajectories in type 2 diabetes patients: a post-hoc analysis of a randomized controlled trial

**DOI:** 10.1186/s12933-022-01482-z

**Published:** 2022-04-04

**Authors:** Johanna M. G. Stultiens, Wiebe M. C. Top, Dorien M. Kimenai, Philippe Lehert, Otto Bekers, Coen D. A. Stehouwer, Adriaan Kooy, Steven J. R. Meex

**Affiliations:** 1 Central Diagnostic Laboratory, Maastricht University Medical Center, P. Debyelaan 25, P.O. Box 5800, 6202 AZ Maastricht, The Netherlands; 2 CARIM School for Cardiovascular Diseases, Maastricht University, Maastricht, The Netherlands; 3 Department of Intensive Care, Care Group Treant, Emmen, The Netherlands; 4 Bethesda Diabetes Research Center, Hoogeveen, The Netherlands; 5 BHF Centre for Cardiovascular Science, University of Edinburgh, Edinburgh, UK; 6 Department of Statistics, Faculty of Economics, Facultés Universitaires Catholiques de Mons, Louvain Academy, Mons, Belgium; 7 Department of Internal Medicine and Cardiovascular Research Institute Maastricht, Maastricht University Medical Center, Maastricht, The Netherlands; 8 Department of Internal Medicine, University Medical Center Groningen, University of Groningen, Groningen, The Netherlands

**Keywords:** Metformin, Troponin, Cardiac, Biomarker, Longitudinal, Cardioprotective, Mechanism

## Abstract

**Background:**

Metformin has favorable effects on cardiovascular outcomes in both newly onset and advanced type 2 diabetes, as previously reported findings from the UK Prospective Diabetes Study and the HOME trial have demonstrated. Patients with type 2 diabetes present with chronically elevated circulating cardiac troponin levels, an established predictor of cardiovascular endpoints and prognostic marker of subclinical myocardial injury. It is unknown whether metformin affects cardiac troponin levels. The study aimed to evaluate cardiac troponin I and T trajectories in patients with diabetes treated either with metformin or placebo.

**Methods:**

This study is a post-hoc analysis of a randomized controlled trial (HOME trial) that included 390 patients with advanced type 2 diabetes randomized to 850 mg metformin or placebo up to three times daily concomitant to continued insulin treatment. Cardiac troponin I and T concentrations were measured at baseline and after 4, 17, 30, 43 and 52 months. We evaluated cardiac troponin trajectories by linear mixed-effects modeling, correcting for age, sex, smoking status and history of cardiovascular disease.

**Results:**

This study enrolled 390 subjects, of which 196 received metformin and 194 received placebo. In the treatment and placebo groups, mean age was 64 and 59 years; with 50% and 58% of subjects of the female sex, respectively. Despite the previously reported reduction of macrovascular disease risk in this cohort by metformin, linear mixed-effects regression modelling did not reveal evidence for an effect on cardiac troponin I and cardiac troponin T levels [− 8.4% (− 18.6, 3.2), p = 0.150, and − 4.6% (− 12, 3.2), p = 0.242, respectively]. A statistically significant time-treatment interaction was found for troponin T [− 1.6% (− 2.9, − 0.2), p = 0.021] but not troponin I concentrations [− 1.5% (− 4.2, 1.2), p = 0.263].

**Conclusions:**

In this post-hoc analysis of a 4.3-year randomized controlled trial, metformin did not exert a clinically relevant effect on cardiac troponin I and cardiac troponin T levels when compared to placebo. Cardioprotective effects of the drug observed in clinical studies are not reflected by a reduction in these biomarkers of subclinical myocardial injury.

*Trial registration* ClinicalTrials.gov identifier NCT00375388.

**Supplementary Information:**

The online version contains supplementary material available at 10.1186/s12933-022-01482-z.

## Background

While its clinical use as a first-line pharmacological means to temper hyperglycemia and increase insulin sensitivity now well exceed 60 years [1], metformin continues to intrigue physicians and scientific researchers from a range of disciplines for its pleiotropic benefits in attenuating complications resulting from (type 2) diabetes and the aging process in general [2].

Findings of the cornerstone long-term UK Prospective Diabetes Study (UKPDS) and the Diabetes Prevention Program Outcome Study are often referenced to substantiate the drug’s cardioprotective effects in populations with newly diagnosed diabetes and prediabetes. The UKPDS, a non-placebo-controlled, open label trial, was the first study showing a reduction of cardiovascular disease (CVD) and cardiovascular (CV) mortality of 40% in obese patients with newly diagnosed type 2 diabetes using metformin compared to controls [3, 4].

The randomized, placebo-controlled trial hyperinsulinemia: the outcome of its metabolic effects (HOME) was designed to investigate whether treatment with metformin, in addition to insulin for glycemic control, could decrease cardiovascular disease in established diabetes patients. As a result, we have previously reported the reduction of macrovascular disease risk observed in the 4.3 year follow-up period [5]. The study design included repeated blood draws, providing opportunity to evaluate (cardiac) biomarkers as a proxy for quantification of cardioprotective effects and to investigate its underlying mechanism.

Specifically, high-sensitivity cardiac troponin I (hs-cTnI) and cardiac troponin T (hs-cTnT) emerged in the last decade as circulating biomarkers with prognostic value. Chronically elevated troponin levels were found to be indicative of subclinical myocardial injury and associated with increased risk of CV outcomes and all-risk mortality in the general population [6, 7] as well as in a range of pathologies [8–12], amongst which (pre)diabetes [13, 14].

With an aim to elucidate the mechanism by which metformin exerts its cardioprotective effects, we hypothesized that metformin treatment may reduce circulating hs-cTnT and hs-cTnI levels. The scope of the current study was to investigate both biomarkers’ trajectories in patients with type 2 diabetes treated with insulin combined either with metformin or placebo.

## Methods

### Study design

**Fig. 1:**
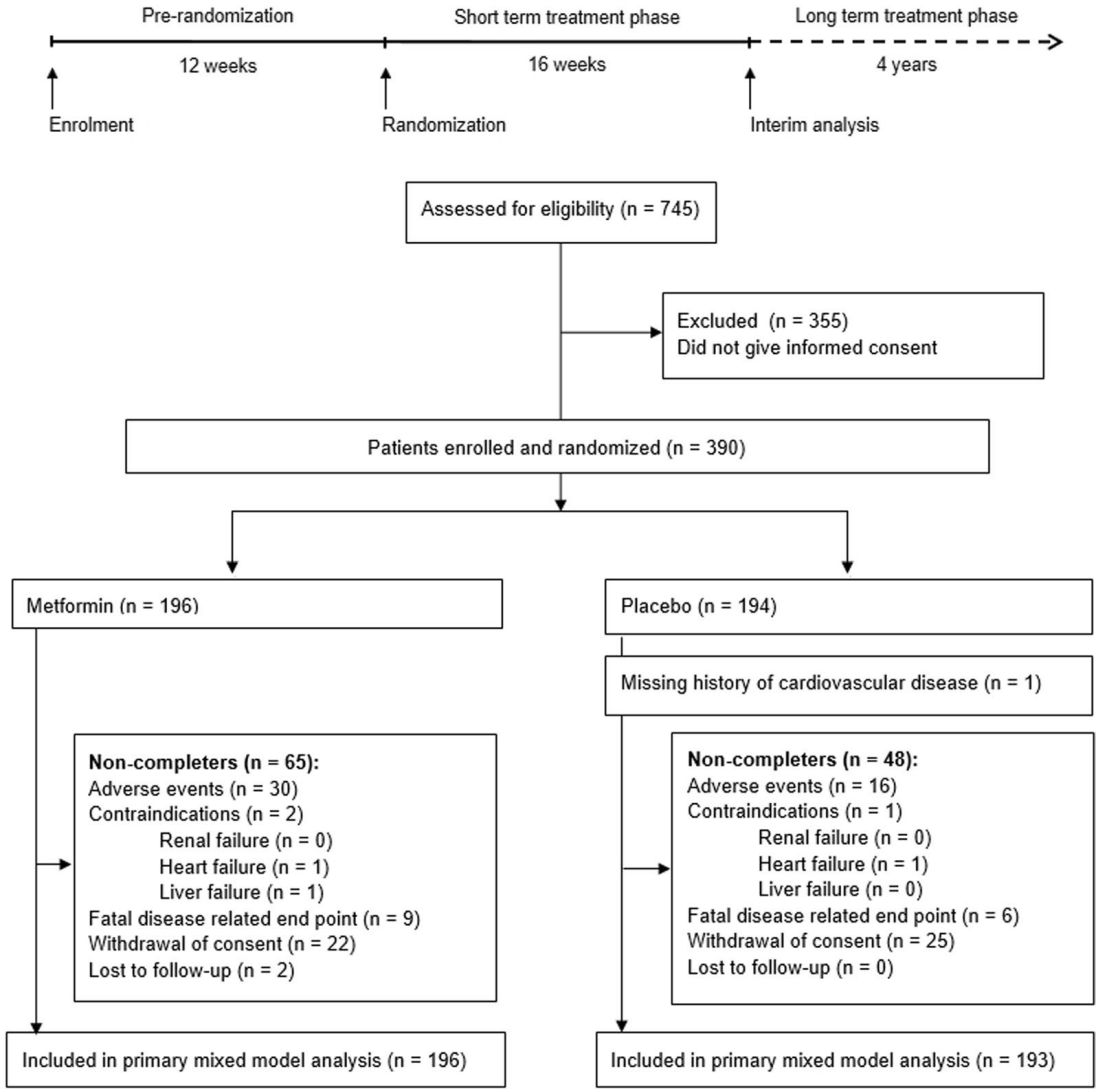
HOME trial schedule and flow diagram

The HOME trial was a randomized trial that included 390 patients with advanced type 2 diabetes mellitus and took place from 1998 to 2002 (ClinicalTrials.gov identifier NCT00375388). The trial was conducted in the outpatient clinics of three non-academic hospitals in the Netherlands: Bethesda General Hospital, Hoogeveen; Isala Diaconessen Hospital, Meppel; and Aleida Kramer Hospital, Coevorden. Refer to Fig. 1 for an overview of the trial design and flow diagram of recruitment and retention of patients.

Participant eligibility criteria and further details are reported elsewhere [15]. Briefly, the HOME trial started with a 12-week pre-randomization phase, in which patients were treated with insulin only and concomitant medication for hypertension and dyslipidemia was discontinued whenever approved by the patient’s physician. Next, patients were randomized to either 850 mg metformin or placebo (1–3 times daily, if tolerated and not contra-indicated) in addition to propagated insulin treatment. No other anti-hyperglycemic agents were used. During this 16-week short-term active treatment phase, target values for blood pressure, urinary albumin-to-creatinine ratio, and plasma low-density lipoprotein (LDL) cholesterol concentrations were less stringent than considered acceptable today, but thought acceptable according to Dutch guidelines during development of the study protocol. Tighter control of these parameters was targeted during the 48-month long-term active treatment phase, using specified medications for treatment of hyperlipidemia and hypertension. An additional requirement for inclusion of each subject’s data in the primary mixed model analysis is that at least one serum sample with corresponding hs-cTnI and hs-cTnT measurements was available.

Study participants provided written informed consent prior to inclusion. The medical ethical committees of the participating hospitals approved the trial protocol. The study was conducted in accordance with Good Clinical Practice (CPMP/ICH/135/95; 1996) and with the Declaration of Helsinki (revised version, 2000).

### Cardiac troponin measurements

Blood samples were drawn at baseline and after 4, 17, 30, 43 and 52 months, and aliquots of serum were stored at − 80 °C until analysis. Serum high-sensitivity cardiac troponin T (Elecsys Troponin T hs STAT assay for Cobas-6000 analyzer, Roche Diagnostics, Basel, Switzerland) and high-sensitivity cardiac troponin I (STAT High Sensitive Troponin-I for Alinity i analyzer, Abbott Diagnostics, Abbott Park, IL, United States) concentrations were measured. The assays were calibrated and quality controlled as intended by the manufacturers. Limit of blank (LoB) and limit of detection (LoD) were 2 ng/L and 3 ng/L, for cardiac troponin T (Roche package insert 08469814500 version 3.0); 1 ng/L and 2 ng/L for cardiac troponin I (Abbott package insert 08P13), respectively. The hs-cTnI assay achieves a 10% coefficient of variation at 4.7 ng/L and a 20% coefficient of variation at 1.3 ng/L. The hs-cTnT assay achieves a 10% coefficient of variation at 13 ng/L and a 20% coefficient of variation at 6.8 ng/L.

### Statistical analysis

Continuous variables were expressed as mean and standard deviation (SD), or median and interquartile range (IQR) for non-parametric variables. Categorical data were reported as n (%). The data and analyses presented concern all randomized patients following the intention-to-treat principle. Cardiac troponin value distributions were skewed and were log-transformed prior to analysis. Upon log-transformation, three influential data points were visually identified (n = 1 for troponin T and n = 2 for troponin I, all three > 250 ng/L), were considered clinical outliers and were therefore not included in the analysis. Baseline and over time comparisons of within-group median cardiac troponin values were done using the paired samples Wilcoxon test. All statistical analysis was performed using R version 3.6.1.

### Primary analysis

We applied a conditional modeling strategy by constructing linear mixed-effects regression models for continuous, within-subject clustered, cardiac troponin I and T data points. The model included fixed effects for treatment assignment (placebo as reference), time as a continuous variable and the interaction between these indicators. Models including random intercept effects only and a combination of random intercept/random slope effects were fitted and compared using the maximum likelihood technique. Identified optimized models were fitted using the restricted maximum likelihood approach, including age, sex, smoking status and cardiovascular history as fixed covariates. Covariance structures for residual errors of longitudinally clustered measures were selected based on the Akaike information criterion (AIC). A sex-stratified analysis was performed by fitting optimized models to each of the sexes individually. Influential data points were identified by determining the Cook’s distance. Optimized models were also fitted to the dataset excluding data points with Cook’s distance > 0.01. Model estimates were reported as antilog values. To facilitate visual interpretation of the effect of metformin on the prevalence of elevated troponin levels, troponin values for which within subject repeated measurements were available at baseline, 4 months and 52 months, were dichotomized at the study’s population sex-specific 75th percentiles. Differences between prevalence of elevated troponin values across treatment groups at each time point were evaluated using the χ^2^ test. In addition, troponin I values were dichotomized using sex-specific risk stratification cut-offs defined by the manufacturer, with values exceeding 4 and 6 ng/L categorized as moderate to high risk for female and male subjects, respectively (package insert version B3P2F0).

### Secondary analyses

We investigated robustness of the primary analysis parameter estimates under departure from the missing at random (MAR) assumption, by conducting supportive analyses on complete cases only (n = 217 for troponin I and n = 220 for troponin T), using multiple imputation (MI) under MAR assumption (MI-MAR) and controlled MI under Missing Not at Random assumption (MI-MNAR). An iterative Markov chain Monte Carlo (MCMC) procedure was performed to generate imputed datasets (*m* = 100), using all variables from the optimized analytical model for MI-MAR and excluding treatment allocation for MI-MNAR. No auxiliary variables were used. Algorithm convergence was assessed by examination of the potential scale reduction factor and using integrated diagnostic plots [16]. Parameter estimates, intraclass correlation coefficient (ICC) and AIC values were averaged following pooling of individual imputation results using Rubin’s rules. Imputed and original data distributions were compared using graphical representations to investigate potential differences.

## Results

### Study patients and baseline characteristics

Following identification of 745 eligible type 2 diabetes patients, a total of 390 subjects enrolled into the trial by providing written informed consent, of which 196 were randomized to receive metformin and 194 to receive placebo (Fig. 1). The trial was completed by 277 subjects (72%) and a total of 46 patients (30 metformin, 16 placebo) discontinued participation due to experienced adverse effects, as previously described [5]. At the final visit, laboratory samples were available for 259 patients (132 placebo and 127 metformin). The actual mean doses in the metformin-treated group were 2163 mg and 2050 mg a day in the short-term and long-term active treatment phases, respectively.

Participants randomized to the metformin treatment arm were slightly older, had a slightly longer duration of diabetes and were less likely to smoke. Distributions of all other clinical factors were balanced between the treatment groups (Table 1).

**Table 1:** Baseline characteristics

	Placebo (n = 191)	Metformin (n = 194)
Sex (female)	95 (50)	113 (58)
Age	59 (11)	64 (10)
Currently smoking (yes)	59 (31)	37 (19)
Body mass index (kg/m^2^)	29.8 (4.8)	29.8 (5.1)
Diabetes duration (years)	12.2 (7.9)	14.2 (8.7)
Cardiovascular history score	0.9 (1.3)	1.2 (1.4)
Systolic blood pressure (mmHg)	160 (24.8)	160 (24.1)
Diastolic blood pressure (mmHg)	85.7 (11.1)	85.4 (11.9)
Blood pressure lowering drugs (yes)	74 (39)	91 (47)
Lipid lowering drugs (yes)	31 (16)	32 (17)
Cardiac Troponin I at baseline	3.0 [2.0, 5.0]	3.6 [2.3, 5.5]
Cardiac Troponin T at baseline	10.0 [6.7, 14.1]	10.2 [7.6, 14.2]
Plasma HbA1c (%, mmol/mol)	7.9 (1.2)	7.9 (1.2)
LDL cholesterol (mmol/l)	3.4 (1.0)	3.6(1.1)
Total cholesterol (mmol/l)	5.5 (1.2)	5.6 (1.3)

Continuous values are presented as mean (standard deviation) or median [interquartile range], as appropriate. Categorical data are presented as absolute numbers (%). Cardiovascular history score is a sum score of cardiovascular events with a range of 0–8

## Metformin and cardiac troponin I and T trajectories in type 2 diabetes patients

A dampening effect of metformin on the course of both troponin I and troponin T [− 8.4% (− 18.6, 3.2) and − 4.6% (− 12, 3.2), respectively] was found, although not statistically significant (p = 0.150 and p = 0.242, respectively). The time-treatment interaction was a statistically significant determinant of troponin T [− 1.6% (− 2.9, − 0.2), p = 0.021] but not troponin I concentrations [− 1.5% (− 4.2, 1.2), p = 0.263], meaning that the favorable effect of metformin on troponin T concentrations increased slightly over time with respect to placebo treatment.

**Fig. 2:**
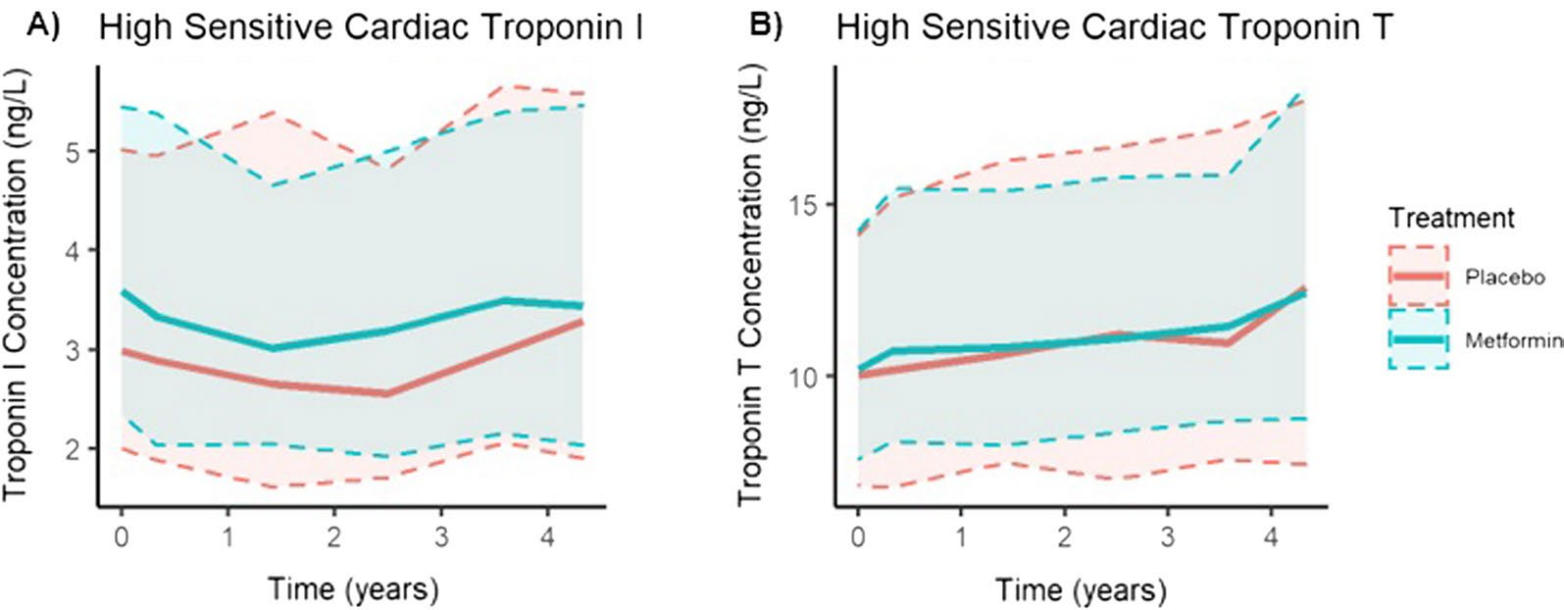
Trajectories of cardiac troponin I (**A**) and T (**B**) with 95% confidence intervals (shaded)

Figure 2 depicts the median concentrations of high-sensitivity cardiac troponin I and T over time. Compared to baseline values, the increase over 52 months of troponin T but not troponin I values was statistically significant in both treatment groups (Table 2).

**Table 2:** Troponin I and Troponin T—baseline, short-term (4 months) and long-term (52 months) comparisons (paired samples Wilcoxon test)

Troponin I concentrations					
Treatment	Time	Observations	Concentration (ng/L) [IQR]	Statistic^a^	p
Placebo	Baseline	188	3.0 [0.6, 25.3]	–	–
	4 months	179	2.9 [0.7, 45.3]	17,078	0.804
	52 months	132	3.3 [0.6, 45.8]	17,926	0.589
Metformin	Baseline	194	3.6 [0.9, 26.2]	–	–
	4 months	179	3.3 [0.7, 24.9]	11,717	0.396
	52 months	126	3.4 [1.0, 31.2]	12,441	0.787

Troponin T concentrations					
Treatment	Time	Observations	Concentration (ng/L) [IQR]	Statistic^a^	p
Placebo	Baseline	191	10.0 [4.2, 30.7]	–	–
	4 months	179	10.2 [4.2, 37.4]	16,311	0.446
	52 months	132	12.6 [4.5, 55.1]	10,243	0.004
Metformin	Baseline	194	10.2 [4.9, 30.8]	–	–
	4 months	179	10.7 [5.3, 32.0]	16,345	0.328
	52 months	127	12.4 [5.2, 44.3]	9932	0.003

*IQR* interquartile range

^a^Test statistic used to calculate the p value

Tables 3 and 4 list the fixed effect estimates of the optimized multilevel model on troponin I and troponin T concentrations, respectively. Treatment effect in the model is defined as the time-independent change in the metformin group relative to the placebo group, including baseline measurements. Optimized models included no within-group correlation for troponin I and first-order autoregressive correlation for troponin T concentrations (estimated *ɸ* reported in Additional file 3: Table S1).

**Table 3:** Random slope/random intercept models of Troponin I

Parameter effect	Optimized model		Cook’s distance	
	Estimate [IQR]	*p*	Estimate [IQR]	*p*
Baseline (ng/L)	0.0 [− 0.3, 0.4]	0.872	− 0.1 [− 0.3, 0.3]	0.771
Treatment (%)	− 8.4 [− 18.6, 3.2]	0.150	− 11 [− 21, 0.2]	0.053
Time effect (%/year)	− 2.7 [− 0.2, − 0.0]	0.005	2.5 [0.5, 4.5]	0.012
Time-treatment interaction (%/year)	− 1.5 [− 4.2, 1.2]	0.263	− 1.0 [− 3.7, 1.7]	0.460
Age (%/year)	2.6 [2.0, 3.2]	< 0.001	2.7 [2.1, 3.3]	< 0.001
Female sex (%)	− 21 [− 30, − 12]	< 0.001	− 21 [− 30, − 12]	< 0.001
Cardiovascular history (%/score unit)	12 [7.4, 17]	< 0.001	13 [− 7.8, 18]	< 0.001

*IQR* interquartile range

**Table 4:** Random slope/random intercept models of Troponin T

Parameter effect	Optimized model		Cook’s distance	
	Estimate [IQR]	*p*	Estimate [IQR]	*p*
Baseline (ng/L)	2.4 [1.7, 3.2]	< 0.001	2.4 [1.7, 3.2]	< 0.001
Treatment (%)	− 4.6 [− 12, 3.2]	0.242	− 4.7 [− 12, 3.1]	0.229
Time effect (%/year)	5.1 [4.1, 6.1]	< 0.001	4.8 [3.9, 5.7]	< 0.001
Time-treatment interaction (%/year)	− 1.6 [− 2.9, − 0.2]	0.021	− 1.4 [− 2.6, − 0.2]	0.023
Age (%/year)	2.3 [1.9, 2.7]	< 0.001	2.3 [1.9, 2.7]	< 0.001
Female sex (%)	− 23.4 [− 29, − 17]	< 0.001	− 23.3 [− 29, − 17]	< 0.001
Cardiovascular history (%/score unit)	5.3 [2.3, 8.4]	0.001	5.2 [2.2, 8.3]	0.001

*IQR* interquartile range

Upon removal of influential data points (Cook’s distance), favorable effects on the course of troponin levels of both metformin treatment and time-treatment interaction were confirmed. The constant treatment effect on troponin I concentrations determined in this subset of observations was not statistically significant [− 11% (− 21, 0.2), p = 0.053], with no change over time [− 1.0% (− 3.7, 1.7), p = 0.460]. The time-treatment effect on troponin T was statistically significant [− 1.4% (− 2.6, − 0.2), p = 0.023], but not the constant treatment effect [− 4.7% (− 12, 3.1), p = 0.229], analogous to the findings obtained without exclusion of influential data points.

Refer to Additional file 3: Table S1 for metrics supporting model comparison. This table includes the ‘null’ model, representing the identified optimized model without any of its independent variables. The introduction of subject-level covariates improves model fit (AIC) and reduces the intra-class correlation coefficient for both troponin I and T values, meaning an increased proportion of the variance is explained by between-subject variance.

A sex-stratified analysis reported in Additional file 3: Table S2 showed that the increase in troponin I concentrations over 52 months was statistically significant in men but not in women and that, while the time-treatment interaction was not a significant determinant of troponin I concentrations in men nor in women, the effect was found to be greater in women. The *p* value for the time-treatment interaction in women neared the commonly used threshold of p < 0.1 for statistical significance of interaction terms. The direct treatment effect on troponin I concentrations was not statistically significant in either sex. For troponin T concentrations, the time-treatment interaction was a significant determinant only in women.

We dichotomized all troponin I and troponin T values using the sex-specific 75th percentile in the study population at baseline as a threshold to define elevated levels of the biomarker (troponin I, women: 4.9 ng/L, men: 5.5 ng/L; troponin T, women: 12.3 ng/L, men: 15.9 ng/L). Additional file 1: Figure S1 shows the relative frequencies of elevated troponin I values (panel A) and troponin T values (panel B) at baseline, 4 months and 52 months, stratified by treatment arm. This approach is based on previous interpretation of the 75th percentile as a threshold to increased CV risk in both patients with stable coronary artery disease and the general population [17, 18]. The incidence of elevated cardiac troponin I levels increased more rapidly for subjects in the placebo group as compared to the metformin group. Differences between the two treatment arms were not statistically significant at any of the time points (results reported in Additional file 3: Table S3). For troponin I, the analysis was repeated using prognostic thresholds reported in the manufacturer’s package insert. Additional file 2: Figure S2 confirms observations reported in Additional file 1: Figure S1 panel A.

### Secondary analyses

Dependent variable missingness was monotone and was 19.5% and 19.3% for cardiac troponin I and cardiac troponin T, respectively. All predictor variable values were available apart from cardiovascular history for one subject (0.25% of data points). Given the missing at random assumption cannot generally be verified and for the current study type discontinuation can be anticipated to be heterogeneous due to dependence on pathology and treatment side-effects, robustness of the primary analysis’ estimates was tested through complete case analysis and multiple imputation (MI) under both missing at random and missing not at random assumptions.

Results from full mixed-effects modelling using only complete cases and multiple imputation under both missing at random and missing not at random missingness mechanisms are reported in Additional file 3: Tables S4 and S5 for troponin I and T, respectively. Trends for constant treatment effect and time-treatment interaction on both cardiac troponins were confirmed, albeit without statistical significance.

## Discussion

The current report covers results of the first post-hoc analysis of a long-term randomized placebo-controlled trial on the effects of metformin on cardiac troponin I and T trajectories. Thorough statistical analysis of hs-cTnI and hs-cTnT levels in the HOME cohort, which previously reported reduced macrovascular disease risk as a result of metformin treatment, did not reveal evidence of a clinically relevant effect of metformin treatment on these cardiac biomarkers, when compared with placebo. Our results corroborate and extend previously published work by Srivastava et al. which investigated short-term effects of metformin on circulation concentrations of cardiac troponin T, reporting no significant effect in a population of relatively recent-onset diabetes [19]. The authors hypothesized that long-term glucose control might lead to reduction in troponin T that was not observed within their 12 week setting.

Baseline troponin levels in the current study cohort were high compared to those in the general population and other type 2 diabetes populations [7, 14, 20]. This can be explained by the relatively long duration of diabetes and cardiovascular comorbidity burden, making the population suitable to investigate risk reduction effects by metformin.

A sex-specific sub-analysis applying the optimized conditional model revealed that the identified trend for the time-treatment interaction was not statistically significant in males. A previous study by Lyons et al. reported altered myocardial glucose metabolism in men but not women treated with metformin, with male subjects exhibiting decreased glucose metabolism and increased myocardial fatty acid (FA) metabolism. In women, metformin was found to reduce myocardial FA metabolism and reduction of left ventricular mass was more pronounced [21]. Female sex was previously reported to be a stronger predictor than obesity for increased FA metabolism, an effect hence counteracted by metformin [22]. Although not clinically relevant, the herein observed sex-specific effect present basis for speculation that metformin induced reduction of myocardial FA metabolism may yield cardioprotective effects specifically in women.

As highlighted previously, few studies were specifically designed to investigate metformin’s effect on cardiovascular endpoints, of which only the HOME study framework allowed for longitudinal biochemical analysis of cardiac circulation markers to quantify its cardioprotective effects and potentially elucidate underlying mechanisms. We have previously investigated the effects of metformin on N-terminal pro B-type natriuretic peptide (NT-proBNP) plasma levels and found no effect when compared to placebo [23]. While metformin’s cardioprotective effects are independent of improved glucose metabolism control [3], our cardiac biomarker studies did not confirm direct involvement of cardiac biomarkers in its mechanism or as a prognostic measure of cardiovascular health improvement. A recent overview of prospective randomized trials evaluating the drug for use in individuals without diabetes mellitus showed largely no effect on cardiovascular outcomes [24]. The exact mechanism for improved cardiovascular health status in a population of overweight patients with (pre)diabetes but not those with other pathologies or healthy controls, remains subject of speculation [3].

## Strengths and limitations

The current study was a post-hoc analysis, and its strengths include the randomized, placebo-controlled, double-blind design, the lengthy follow-up period during which serum samples were collected frequently and a direct comparison of cardiac troponin I and T. Also, given the study was conducted in a non-academic setting, patient recruitment is reflective of actual peripheral diabetes management.

Our study also has some limitations. First, imbalance between treatment groups after randomization. Although the mixed model analysis approach taken is robust against baseline differences, we cannot completely rule out residual confounding and the imbalance hampered straightforward data visualization and interpretation. Second, the power analysis of the HOME trial was based on the studies’ original primary micro- and macrovascular endpoints [15]; nevertheless the statistically significant effects found were deemed not clinically relevant and we therefore argue that at the current state of the art for high sensitivity troponin detection combined with known biological within-subject variation, it is likely that inclusion of more subjects would not impact statistical significance [9, 25]. Third, all participants were middle-aged, Caucasian, insulin-treated individuals with a relatively long duration of type 2 diabetes and the findings cannot be extrapolated to patients with other ethnicities or different medical background [26]. Lastly, no data exists documenting troponin I and T stability after approximately 20 years. Concerns have been expressed regarding interpretation of cardiac troponin data originating from samples stored long-term at − 80 °C as analyte degradation presents a potential source of noise [27, 28].

## Conclusions

In conclusion, our findings show that in patients with long-lasting type 2 diabetes that are intensively treated with insulin, the concomitant use of metformin does not affect circulating cardiac troponin I and T as compared to placebo when tested in a trial cohort that previously reported reduction in cardiovascular disease associated with the drug. Metformin exerts its cardioprotective effects in a way that does not alter cardiac troponin levels, a finding relevant to elucidate its exact mechanism in the future.

## Supplementary Information

**Additional file 1: Figure S1.** Prevalence of Troponin T and I levels > 75th percentile.

**Additional file 2: Figure S2.** Prevalence of Troponin I levels exceeding the manufacturer’s low risk threshold.

**Additional file 3: Table S1.** Troponin I and Troponin T linear mixed-effects model metrics. **Table S2.** Sex-stratified mixed models of Troponin I and Troponin T. **Table S3.** Prevalence of elevated cardiac troponins in placebo vs. metformin. **Table S4.** Random slope/random intercept models as sensitivity analysis for Troponin I. **Table S5.** Random slope/random intercept models as sensitivity analysis for Troponin T.

## Data Availability

The datasets used and/or analysed during the current study are available from the corresponding author on reasonable request.

## Abbreviations

AIC: Akaike information criterion
CV: Cardiovascular
CVD: Cardiovascular disease
GLP-1-RA: Glucagon-like peptide 1 receptor antagonist
hs-cTnI: High-sensitivity cardiac troponin I
hs-cTnT: High-sensitivity cardiac troponin T
HOME (trial): Hyperinsulinemia: the outcome of its metabolic effects
ICC: Intraclass-correlation coefficient
IQR: Interquartile range
LDL: Low-density lipoprotein
LoB: Limit of blank
LoD: Limit of detection
MAR: Missing at random
MI: Multiple imputation
MI-MAR: Multiple imputation under missing at random assumption
MI-MNAR: Multiple imputation under missing not at random assumption
NT-proBNP: N-terminal pro B-type natriuretic peptide
SD: Standard deviation
SGLT2-I: Sodium/glucose cotransporter 2 inhibitor
UKPDS: United Kingdom Prosepctive Diabetes Study
