# Supplementary figures and images for: Metformin and high-sensitivity cardiac troponin I and T trajectories in type 2 diabetes patients: a post-hoc analysis of a randomized controlled trial
Source: Cardiovasc Diabetol. 2022 Apr 4;21:49. doi: 10.1186/s12933-022-01482-z (PMC8981770; doi:10.1186/s12933-022-01482-z)

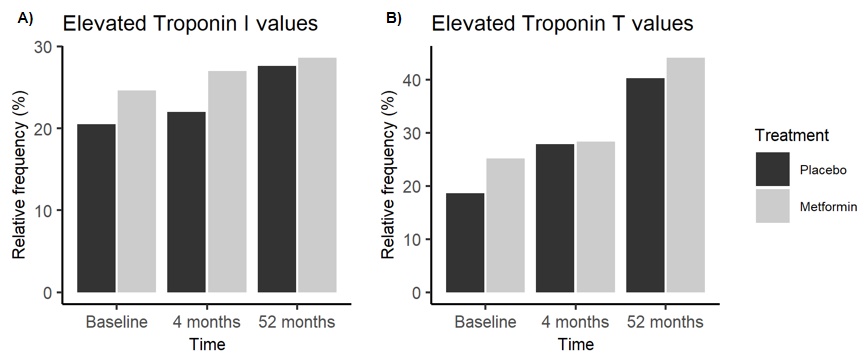

Supplement: Supplementary file 1 — Additional file 1: Figure S1. Prevalence of Troponin T and I levels > 75th percentile. [file 12933_2022_1482_MOESM1_ESM.jpg]

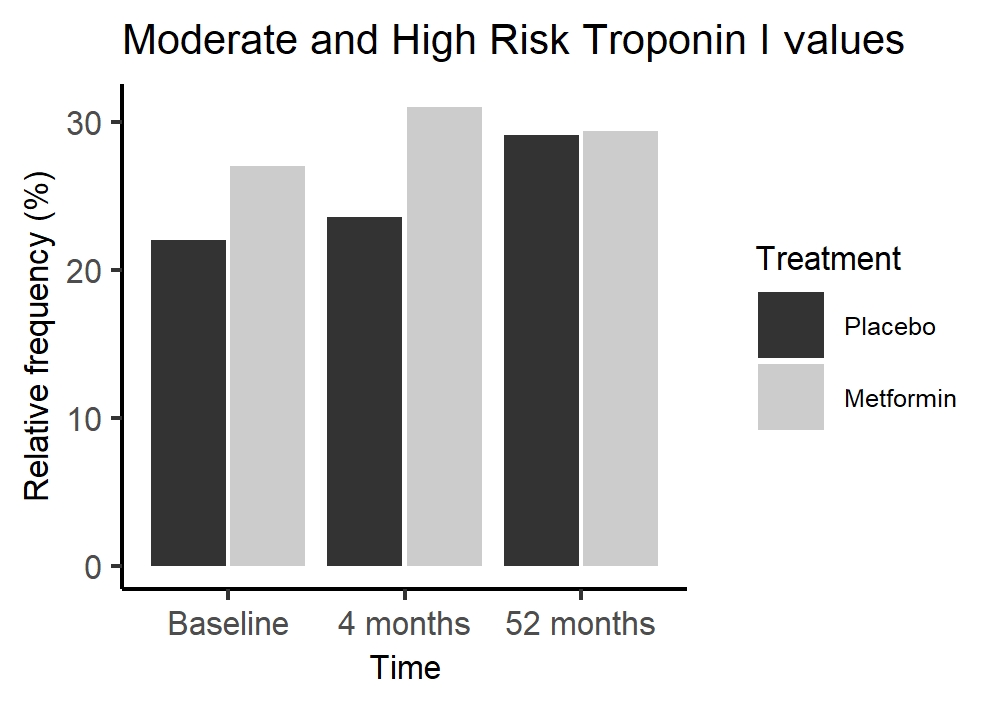

Supplement: Supplementary file 2 — Additional file 2: Figure S2. Prevalence of Troponin I levels exceeding the manufacturer’s low risk threshold. [file 12933_2022_1482_MOESM2_ESM.jpg]
