# Supplementary material for: Metformin and high-sensitivity cardiac troponin I and T trajectories in type 2 diabetes patients: a post-hoc analysis of a randomized controlled trial
Source: Cardiovasc Diabetol. 2022 Apr 4;21:49. doi: 10.1186/s12933-022-01482-z (PMC8981770; doi:10.1186/s12933-022-01482-z)
Supplement: Supplementary file 3 — Additional file 3: Table S1. Troponin I and Troponin T linear mixed-effects model metrics. Table S2. Sex-stratified mixed models of Troponin I and Troponin T. Table S3. Prevalence of elevated cardiac troponins in placebo vs. metformin. Table S4. Random slope/random intercept models as sensitivity analysis for Troponin I. Table S5. Random slope/random intercept models as sensitivity analysis for Troponin T. [file 12933_2022_1482_MOESM3_ESM.docx]

Metformin and high-sensitivity cardiac troponin I and T trajectories in type 2 diabetes patients – a post-hoc analysis of a randomized controlled trial

Johanna MG Stultiens, MSc, Wiebe MC Top, MD, Dorien M Kimenai, PhD, Philippe Lehert, PhD, Otto Bekers, PhD, Coen DA Stehouwer, MD, PhD, Adriaan Kooy, MD, PhD, Steven JR Meex, PhD

Additional file

Figure S1: Prevalence of Troponin T and I levels > 75^th^ percentile

Figure S2: Prevalence of Troponin I levels exceeding the manufacturer’s low risk threshold

Table S1: Troponin I and Troponin T linear mixed-effects model metrics

| **Troponin I – Model metrics** | | | |
| --- | --- | --- | --- |
|  | ‘Null’ Model  (n=390) | Optimized Model  (n = 389) | Cook’s Distance  (n = 389) |
| ICC | 0.821 | 0.765 | 0.731 |
| AIC | 2289.9 | 2196.8 | 2432.5 |
|  | | | |
| **Troponin T – Model metrics** | | | |
|  | ‘Null’ Model  (n=390) | Optimized Model  (n = 389) | Cook’s Distance  (n = 389) |
| ICC | 0.875 | 0.808 | 0.830 |
| AIC | 242.3 | 6.46 | -263.8 |
| AR(1) Phi [IQR] | 0.05 [0.02, 0.12] | 0.05 [0.02, 0.15] | 0.07 [0.03, 0.17] |

Abbreviations: IQR = interquartile range

Table S2: Sex-stratified mixed models of Troponin I and Troponin T

| **Troponin I** | Men (n = 177) | | Women (n = 212) | |
| --- | --- | --- | --- | --- |
| **Parameter effect** | Estimate [IQR] | *p* | Estimate [IQR] | *p* |
| Baseline (ng / L) | 0.0 [-0.4, 0.7] | 0.969 | -0.1 [-0.4, 0.3] | 0.540 |
| Treatment (%) | -10 [-26, 8.9] | 0.270 | -6.9 [-20, 7.8] | 0.339 |
| Time effect (%/year) | 3.8 [0.7, 7.0] | 0.017 | 1.5 [-0.6, 3.6] | 0.162 |
| Time-treatment interaction (%/year) | 0.0 [-4.5, 4.7] | 0.998 | -2.1 [-4.9, 0.7] | 0.137 |
| Age (%/year) | 2.6 [1.6, 3.5] | < 0.001 | 2.5 [1.8, 3.3] | < 0.001 |
| Cardiovascular history (%/score unit) | 18 [9.7, 27] | < 0.001 | 8.1 [2.7, 14] | < 0.001 |
| **Troponin T** | Men (n = 177) | | Women (n = 212) | |
| **Parameter effect** | Estimate [IQR] | *p* | Estimate [IQR] | *p* |
| Baseline (ng / L) | 2.4 [1.4, 3.7] | < 0.001 | 1.6 [0.9, 2.6] | < 0.001 |
| Treatment (%) | -8.0 [-18, 3.7] | 0.171 | -1.6 [-11, 9.3] | 0.761 |
| Time effect (%/year) | 5.2 [3.7, 6.7] | < 0.001 | 4.8 [3.6, 6.0] | < 0.001 |
| Time-treatment interaction (%/year) | -1.3 [-3.4, 0.8] | 0.234 | -1.7 [-3.3, -0.1] | 0.037 |
| Age (%/year) | 2.3 [1.7, 2.9] | < 0.001 | 2.2 [1.7, 2.7] | < 0.001 |
| Cardiovascular history (%/score unit) | 7.1 [2.2, 12.2] | 0.005 | 4.2 [0.4, 8.1] | 0.029 |

Abbreviations: IQR = interquartile range

Table S3: Prevalence of elevated cardiac troponins in placebo vs. metformin

| Troponin I concentrations > 75th percentile (n = 253) | | |
| --- | --- | --- |
| **Visit** | χ^2^ statistic | p value |
| Baseline | 0.404 | 0.525 |
| At 4 months | 0.588 | 0.443 |
| At 52 months | 0.002 | 0.969 |
|  |  |  |
| Troponin T concentrations > 75th percentile (n = 256) | | |
| **Visit** | χ^2^ statistic | p value |
| Baseline | 1.26 | 0.261 |
| At 4 months | 0 | 1 |
| At 52 months | 0.237 | 0.627 |

Table S4: Random slope / random intercept models as sensitivity analysis for Troponin I

|  | Complete case  (n = 217) | | MI (MAR)  (n = 389, m = 100) | | MI (MNAR)  (n = 389, m = 100) | |
| --- | --- | --- | --- | --- | --- | --- |
| **Parameter effect** | Estimate [IQR] | *p* | Estimate [IQR] | *p* | Estimate [IQR] | *p* |
| Baseline (ng / L) | 0.0 [-0.4, 0.5] | 0.868 | 0.0 [-0.3, 0.4] | 0.981 | 0.0 [-0.3, 0.4] | 0.977 |
| Treatment (%) | -8.0 [-21, 7.2] | 0.285 | -10 [-20, 1.5] | 0.085 | -10 [-20, 1.5] | 0.086 |
| Time effect (%/year) | 2.3 [0.1, 4.5] | 0.040 | 2.3 [0.3, 4.5] | 0.025 | 2.3 [0.1, 4.2] | 0.043 |
| Time-treatment interaction (%/year) | -0.8 [-3.8, 2.3] | 0.606 | -1.3 [-4.2, 1.7] | 0.396 | -0.8 [-3.5, 2.0] | 0.566 |
| Age (%/year) | 2.7 [1.9, 3.4] | < 0.001 | 2.6 [2.0, 3.2] | < 0.001 | 2.6 [2.0, 3.2] | < 0.001 |
| Female sex (%) | -26 [-36, -15] | < 0.001 | -21 [-30, -11] | < 0.001 | -21 [-30, -11] | < 0.001 |
| Cardiovascular history (%/score unit) | 10 [4.0, 17] | 0.001 | 13 [7.8, 18] | < 0.001 | 13 [7.7, 18] | < 0.001 |

Abbreviations: IQR = interquartile range

Table S5: Random slope / random intercept models as sensitivity analysis for Troponin T

|  | Complete case  (n = 220) | | MI (MAR)  (n = 389, m = 100) | | MI (MNAR)  (n = 389, m = 100) | |
| --- | --- | --- | --- | --- | --- | --- |
| **Parameter effect** | Estimate [IQR] | *p* | Estimate [IQR] | *p* | Estimate [IQR] | *p* |
| Baseline (ng / L) | 2.5 [1.7, 3.7] | < 0.001 | 2.3 [1.6, 3.2] | < 0.001 | 2.3 [1.6, 3.1] | < 0.001 |
| Treatment (%) | -4.4 [-13, 5.5] | 0.367 | -5.4 (-13, 2.4] | 0.171 | -5.4 (-13, 2.4] | 0.173 |
| Time effect (%/year) | 4.2 [3.2, 5.3] | < 0.001 | 5.0 (3.7, 6.3] | < 0.001 | 4.8 (3.5, 6.0] | < 0.001 |
| Time-treatment interaction (%/year) | -0.8 [-2.2, 0.6] | 0.277 | -1.5 [-3.2, 0.2] | 0.549 | -1.1 [-2.8, 0.5] | 0.197 |
| Age (%/year) | 2.2 [1.7, 2.7] | < 0.001 | 2.3 [1.9, 2.7] | < 0.001 | 2.3 [1.9, 2.7] | < 0.001 |
| Female sex (%) | -25 [-32, -17) | < 0.001 | -23 [-29, -17) | < 0.001 | -23 [-29, -17) | < 0.001 |
| Cardiovascular history (%/score unit) | 5.3 [1.3, 9.4] | 0.010 | 5.1 [2.1, 8.3] | 0.001 | 5.2 [2.1, 8.3] | 0.001 |

Abbreviations: IQR = interquartile range
